# Tendencia temporal y factores asociados al parto prematuro en Chile, 1992-2018

**DOI:** 10.18294/sc.2023.4203

**Published:** 2023-02-01

**Authors:** Carol Toro-Huerta, Carolina Vidal, Luis Araya-Castillo

**Affiliations:** 1 Magíster en Políticas Públicas. Instituto de Salud Pública, Universidad Andrés Bello. Santiago, Chile. carol.toro@unab.cl Universidad Andrés Bello Instituto de Salud Pública Universidad Andrés Bello Santiago Chile carol.toro@unab.cl; 2 Magíster en Salud Pública. Instituto de Salud Pública, Universidad Andrés Bello. Santiago, Chile. krolina.vidalg@gmail.com Universidad Andrés Bello Instituto de Salud Pública Universidad Andrés Bello Santiago Chile krolina.vidalg@gmail.com; 3 PhD in Mangement Sciences. Decano, Facultad de Ingeniería y Empresa, Universidad Católica Silva Henríquez, Santiago de Chile, Chile. larayac@ucsh.cl Universidad Católica Silva Henríquez Facultad de Ingeniería y Empresa Universidad Católica Silva Henríquez Santiago de Chile Chile larayac@ucsh.cl

**Keywords:** Parto Prematuro, Edad Materna, Salud Materna, Salud Infantil, Chile

## Abstract

Se realizó un estudio analítico con base en los registros poblacionales de nacimientos en Chile, obtenidos del Departamento de Estadística e Información en Salud (DEIS), con el objetivo de evaluar la tendencia temporal de los partos prematuros en Chile en el periodo 1990-2018, asociado a la edad de la madre. Los resultados muestran que, para el año 1992, la tasa de parto prematuro fue del 5,0%, aumentando a 7,2% en 2018. El promedio del porcentaje del cambio anual (PPCA) fue de 1,44. Los grupos etarios extremos -menor o igual de 19 años y 35 y más años- fueron los que presentaron las tasas de parto prematuro más altas, tanto al inicio y como al término del periodo, siendo este último grupo el que mostró una menor disminución al inicio del periodo (1992-1995), con porcentaje de cambio anual (PCA) de -3,00. Para ambos grupos, la probabilidad de un parto prematuro fue mayor respecto del grupo de 20 a 34 años. Chile, presenta uno de los mejores indicadores de salud materna e infantil para la región; no obstante, dada la actual postergación de la maternidad, deben vigilarse las repercusiones asociadas, dentro de ellas un nacimiento prematuro.

## INTRODUCCIÓN

La Organización Mundial de la Salud (OMS) define el nacimiento prematuro como aquel que ocurre antes de las 37 semanas de gestación. La prematuridad continúa siendo un importante problema de salud pública en muchos países, pues sus consecuencias son múltiples^1^. Los recién nacidos prematuros poseen un mayor riesgo de morbilidad y mortalidad durante el periodo neonatal y de complicaciones neurológicas y respiratorias a largo plazo, además presentan en la adultez un mayor riesgo de enfermedades crónicas y de desórdenes psiquiátricos^2^. Por otra parte, en las madres de niños prematuros, se observa una tendencia más elevada a presentar depresión y estrés postraumático respecto de aquellas que tuvieron un parto de término^3^.

La prevalencia del parto prematuro ha aumentado durante las últimas décadas en la mayoría de los países que presentan registros confiables^4^. Con un estimado de 15 millones de nacimientos prematuros para el año 2014, la tasa global de parto prematuro aumentó de 9,8% en el año 2000 a 10,6% para el año 2014, asimismo se observaron variaciones de acuerdo con la región, con tasas de 13,4% y 8,7% para África del norte y Europa respectivamente^5^.

Las causas del parto prematuro son diversas, tales como patologías obstétricas, infecciones y edad materna igual o superior a los 35 años^6^. La evidencia señala que la edad materna avanzada está asociada a complicaciones obstétricas como diabetes gestacional, hipertensión y preeclampsia, así como a complicaciones fetales como restricción de crecimiento intrauterino y prematuridad^7^. 

En las últimas décadas, mujeres de países de altos y medianos ingresos han mostrado una tendencia a postergar la gestación. En EEUU, la edad promedio de las mujeres para tener su primer hijo, aumentó de 24,2 en el año 2000 a 26,3 en el año 2014. Además, el primer nacimiento en mujeres de 35 a 39 años aumentó en un 64% y en mujeres de 40 a 44 aumentó en un 230% para el mismo periodo^8^.

Chile ha presentado cambios en la tendencia de nacimientos por edad. El estudio de López en 2015 muestra que la proporción de nacimientos en mujeres de 35 años y más pasó de 10,6% en 1991 a 16,6% en 2012. Además, la tasa de parto prematuro aumentó de 4,7% a 6,4% en mujeres de 34 años y más durante el mismo periodo, lo que corresponde a un aumento de un 29%^9^. Adicionalmente, López Orellana muestra un *odds ratio* (OR) de 1,68 (IC95% [1,66; 1,70]) para nacimiento prematuro en madres de 35 años y más, comparado con madres de 20 a 29 años, ajustado por educación, estado civil y paridad^9^.

Reducir las tasas de parto prematuro es un desafío mundial para alcanzar el Objetivo de Desarrollo Sostenible 3: “garantizar una vida sana y promover el bienestar de todos a todas las edades”^10^. Si bien Chile presenta buenos indicadores de salud materna e infantil, producto de políticas aplicadas en décadas pasadas, el lento desarrollo de medidas preventivas para el parto prematuro, la postergación de la maternidad y las nuevas tecnologías utilizadas en las unidades neonatales deben considerarse para enfrentar el problema. Para ello, es necesario contar con un diagnóstico regional actualizado respecto de sus causas y población de riesgo que permita dirigir estrategias y políticas. 

Conforme lo anterior señalado, se plantea como objetivo de estudio, evaluar la tendencia temporal de los partos prematuros en Chile, en el periodo 1990-2018, asociado a la edad de la madre. A la fecha no existen estudios actualizados que abarquen información por un periodo prolongado de años y que den cuenta del comportamiento del parto prematuro relacionado a la edad materna.

## MATERIAL Y MÉTODO

Se realizó un estudio analítico de los registros poblacionales de nacimientos en Chile, obtenidos del Departamento de Estadística e Información en Salud (DEIS). Los registros contienen la información de todos los nacimientos ocurridos en Chile durante el periodo 1992-2018, los cuales son extraídos de forma rutinaria de los certificados de nacimiento^11^. El conjunto de datos involucra características sociodemográficas y de salud de niños y niñas, demografía de los padres y factores maternos asociados a los nacimientos. 

La prematuridad es definida por la Organización Mundial de la Salud (OMS) como el nacimiento que ocurre antes de completarse las 37 semanas o antes de 259 días de gestación, desde el primer día del último periodo menstrual. Se subdivide en prematuros extremos (<28 semanas), muy prematuros (28 a <32 semanas) y prematuros moderados o tardíos (32 a <37 semanas)^4^^,^^12^. Se excluyeron los partos múltiples y los nacimientos que no corresponden a la definición de nacidos vivos (menores de 22 semanas de edad gestacional y/o con menos de 500 gramos).

Se calcularon las tasas anuales de parto prematuro en los nacidos vivos únicos junto con las tasas específicas según edad de la madre de acuerdo con tres categorías: menor o igual de 19 años, 20 a 34, y 35 y más años. Para identificar el momento en que se produjeron cambios significativos en la tendencia (y la observada en el intervalo), durante el periodo 1992-2018, se construyó un modelo de regresión de *joinpoint* (regresión de Poisson segmentada). Este modelo identifica el momento en que se producen cambios estadísticamente significativos en la tendencia y, además, estima la tendencia observada en dicho intervalo mediante el porcentaje de cambio anual (PCA), el cual es una forma de caracterizar las tendencias de las tasas a lo largo del tiempo. En función de la cantidad de registros para el análisis, se probó con un ajuste con un máximo de cinco *joinpoints*. Lo que hace el programa es elegir el menor número de *joinpoints*, de manera que, si se añade un *joinpoint* más, la mejora no es estadísticamente significativa. El programa comienza con el número mínimo de puntos de unión (0) para ir añadiendo un nuevo punto de unión (*jointpoint*) mediante pruebas de permutación hasta seleccionar el modelo final^13^.

Respecto a los grupos de edad de las madres, se evaluó la tendencia según el nivel educacional alcanzado de la madre: bajo (educación básica), medio (educación media), alta (educación superior). Para cada uno de estos grupos, mediante el programa *joinpoint*, se estimó el cambio promedio del porcentaje de cambio anual, el cual corresponde a una medida resumida de la tendencia durante el periodo de estudio. 

Para evaluar la asociación de la edad materna y el parto prematuro, se utilizó un modelo de regresión logística y se calcularon *odds ratio* (OR) y sus intervalos de confianza al 95% (IC95%). Las variables de ajuste que se incluyeron fueron sexo del recién nacido, año de nacimiento (para evaluar la tendencia), nivel educacional, actividad y área de residencia de la madre. 

Para este estudio se utilizaron registros de estadísticas vitales, las cuales son de uso y acceso público, obtenidos del Ministerio de Salud de Chile, por lo que los registros son confidenciales y anónimos según lo dispuesto en la Ley 17374, artículo 29^14^. 

## RESULTADOS

De un total de 6.679.532 nacidos vivos registrados durante el periodo 1992-2018, 141.917 se excluyeron y 6.537.615 nacidos vivos únicos se incluyeron en el análisis. Dentro de estos, 3.250 corresponden a partos < de 500 g o < de 22 semanas, 124.290 corresponden a partos múltiples y 14.377 a datos perdidos.

**Figura 1:**
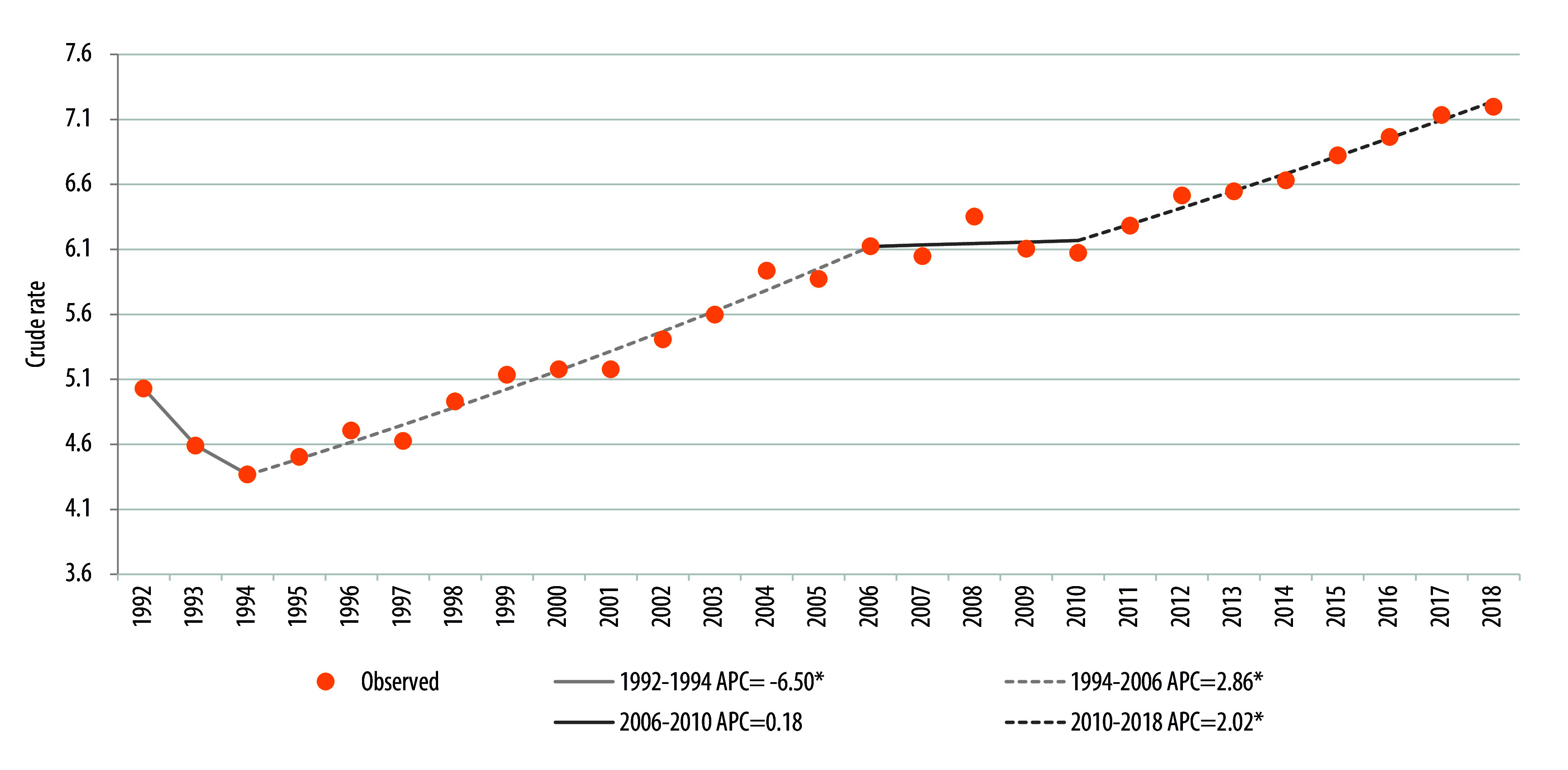
Evolución temporal y puntos de cambio en la tasa de parto prematuro. Chile, 1992-2018.

La Figura 1 muestra los cambios en la tendencia de parto prematuro durante el periodo. Para el año 1992 la tasa fue del 5,0%, mientras que, para el año 2018, fue del 7,2%. El promedio del porcentaje del cambio anual (PPCA) fue de 1,44 (IC95% [0,87; 2,01]). Se observaron tres cambios (*joinpoints*) en la tendencia. En el primer segmento (periodo 1992-1994), se observó una disminución en la tendencia con un PCA -6,50 (IC95% [-11,33; -1,40]). En el segundo periodo 1994-2006, se observó un aumento con un PCA de 2,86 (IC95% [2,49; 3,24]). Le siguen otros dos periodos de aumento: entre 2006 y 2010, con un PCA 0,18 (IC95% [-2,29; 2,72]), y entre 2010 y 2018, con un PCA 2,02 (IC95% [1,48; 2,57]). Todos los periodos señalados presentaron cambios estadísticamente significativos, excepto el observado entre 2006 y 2010.

Fuente: Elaboración propia. PCA= Porcentaje del cambio anual. *Indica que el porcentaje de cambio anual es significativamente diferente de cero, con un nivel alfa=0,05

**Figura 2:**
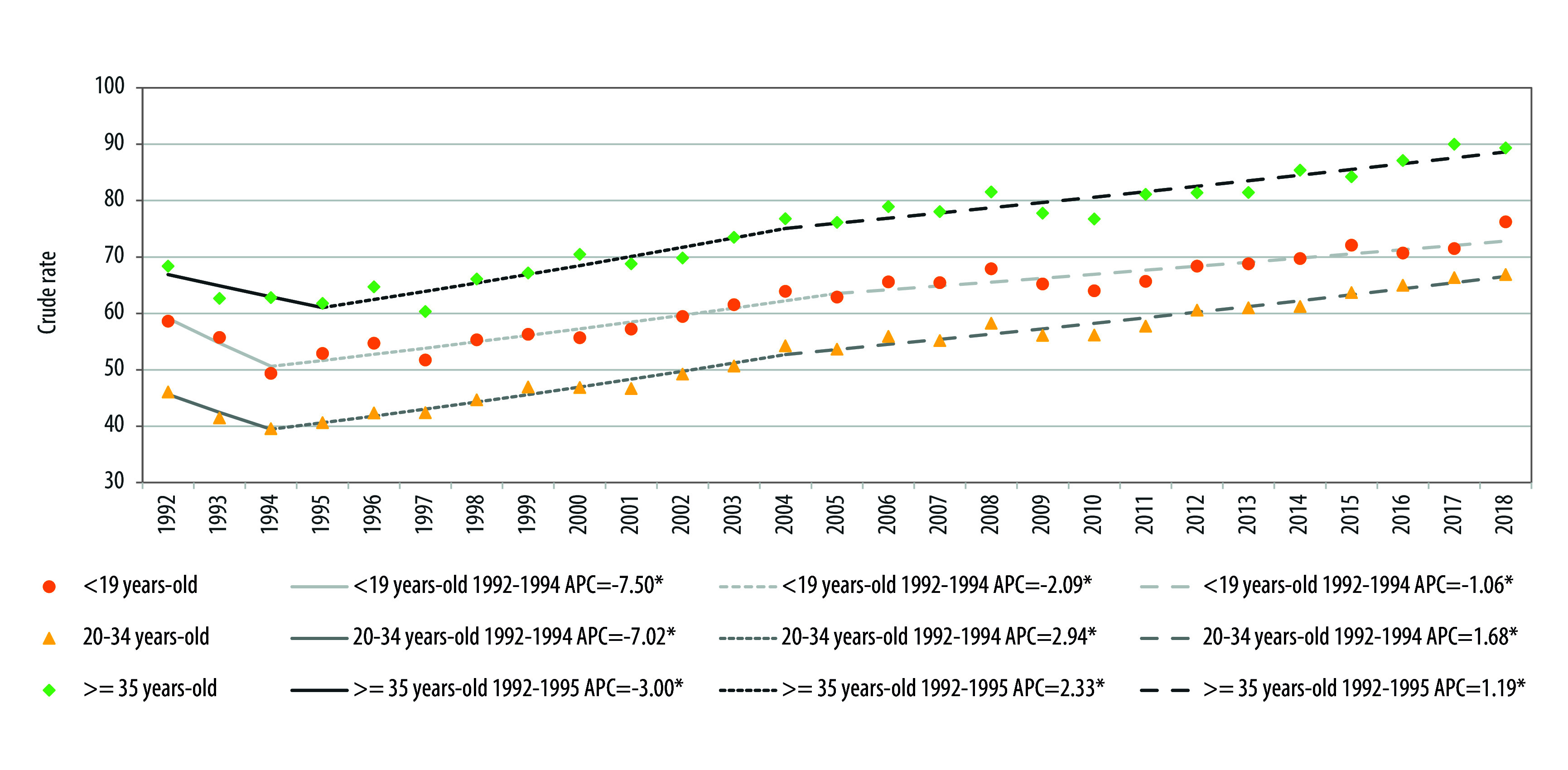
Evolución temporal y puntos de cambio en la tasa cruda de partos prematuros según edad de la madre. Chile, 1992-2018.

La Figura 2 y la Tabla 1 muestran los cambios en la tendencia de parto prematuro de acuerdo a la edad de la madre. Para el grupo de edad menor o igual a 19 años se observaron 2 *joinpoints*. En el primer periodo entre 1992 y 1994 se observó una disminución en el parto prematuro, con un PCA de -7,50 (IC95% [-14,36; -0,09]), el segundo periodo entre 1994 a 2005 y el tercer periodo entre 2005 a 2018, mostraron un aumento con PCA de 2,09 (IC95% [1,47; 2,71]), y 1,06 (IC95% [0,61; 1,50]), respectivamente, ambos estadísticamente significativos.

Fuente: Elaboración propia. PCA= Porcentaje del cambio anual. *Indica que el porcentaje de cambio anual es significativamente diferente de cero, con un nivel alfa=0,05.

**Tabla 1:** Descripción del número de *joinpoints*, porcentaje de cambio anual e intervalo de confianza del 95%, según edad de la madre. Chile, 1992-2018.

Grupo de edad	Joinpoint	Punto inferior	Punto superior	PCA	IC95%
<19 años	2	1992	1994	-7,50*	-14,36; -0,09
		1994	2005	2,09*	1,47; 2,71
		2005	2018	1,06*	0,61; 1,50
20-34 años	2	1992	1994	-7,02*	-12,23; -1,43
		1994	2004	2,94*	2,37; 3,52
		2004	2018	1,68*	1,41; 1,94
> = 35 años	2	1992	1995	-3,00	-7,44; 1,66
		1995	2004	2,33*	1,36; 3,30
		2004	2018	1,19*	0,84; 1,54

Fuente: Elaboración propia. PCA= Porcentaje del cambio anual. *Significativamente diferente de cero, con un nivel alfa= 0,05

En el grupo etario de 20 a 34 años se observaron 2 *joinpoints* en periodos y magnitudes similares a los descritos en el tramo etario anterior, con una disminución en el PCA -7,02 y luego 2 aumentos en los periodos siguientes, con PCA de 2,94 entre 1994 y 2004, y de 1,68 entre 2004 y 2018.

Por último, en el grupo etario de 35 y más años, también se observaron 2 *joinpoints*. Durante el primer periodo de 1992 a 1995 hubo una disminución, pero de menor magnitud que la observada en el mismo periodo para los grupos etarios anteriores, con un PCA de -3,00. Luego 2 aumentos: de 1995 a 2004, con un PCA de 2,33, y de 2004 a 2018, con un PCA de 1,19.

El subanálisis que incorpora el promedio PCA de parto prematuro, entre los años 1992 y 2018 según nivel educacional materno (Tabla 2), muestra un aumento del promedio de PCA en todos los niveles educacionales para cada uno de los tramos de edad, siendo en el nivel de educación básica levemente superior. En concordancia con los resultados antes expuestos, se observa que las mujeres de 35 o más años, son las que presentan las tasas más altas y, además, dentro de este grupo, la tendencia ha sido levemente mayor en aquellas mujeres con el nivel educacional más bajo, con un promedio PCA de 2,03 (IC95% [1,73 - 2,33]).

**Tabla 2:** Promedio del porcentaje del cambio anual de la tasa cruda de parto prematuro e intervalo de confianza del 95%, según edad y nivel educacional de la madre. Chile, 1992-2018.

Variable	Tasa cruda		Promedio PCA	IC 95%
	1990	2018		
<19 años				
Educación básica	6,09	8,18	1,31	0,39 - 2,24
Educación media	5,60	7,59	0,89	0,18 - 1,60
Educación superior	5,61	6,29	0,84	0,14 - 1,54
20-34 años				
Educación básica	4,62	7,57	2,07	1,49 - 2,65
Educación media	4,57	6,81	1,61	0,99 - 2,23
Educación superior	4,32	6,42	2,00	1,62 - 2,30
> = 35 años				
Educación básica	6,70	9,88	2,03	1,73 - 2,33
Educación media	7,03	9,43	1,56	1,35 - 1,77
Educación superior	6,40	8,37	1,24	1,00 - 1,49

Fuente: Elaboración propia. PCA= Porcentaje del cambio anual. IC95%= Intervalo de confianza del 95%

El modelo de regresión logística (Tabla 3) muestra que la probabilidad de un parto prematuro en mujeres de 35 años y más es de 1,44 (IC95% [1,43; 1,45]) respecto del grupo de 20 a 34 años, ajustando por sexo y año de nacimiento del recién nacido, nivel educacional, actividad y área de residencia de la madre. De igual modo se observó que esta probabilidad aumentaba en madres adolescentes (<= a 19 años).

**Tabla 3:** Distribución de *odds ratio* e intervalo de confianza del 95% de la probabilidad de parto prematuro, según variables sociodemográficas. Chile, años 1992-2018.

Variables	Distribución de los datos		Parto prematuro > = 22 semanas y < 37 semanas			
	n	%	n	%	OR	IC95%
Edad de la madre						
<19 años	940.485	14,4	57.808	6,1	1,19	1,18 – 1,20
20 a 34 años^1^	4.598.130	70,3	241.365	5,2	1,00	-
35 años o más	998.215	15,3	75.554	7,6	1,44	1,43 – 1,45
Sexo del recién nacido						
Varón	3.342.773	51,1	207.505	6,2	1,20	1,19 – 1,20
Mujer^1^	3.194.737	48,9	167.213	5,2	1,00	-
Nivel educacional madre						
Educación superior^1^	1.498.242	22,9	89.228	6,0	1,00	-
Educación media	3.714.865	56,8	209.952	5,7	1,01	1,00 – 1,02
Educación básica	1.299.694	19,9	72.937	5,6	1,07	1,05 – 1,08
Ninguna	17.465	0,3	1.218	7,0	1,39	1,31 – 1,47
Ocupación						
Inactivo^1^	4.405.885	67,4	247.841	5,6	1,00	-
Activo	2.110.988	32,3	125.466	5,9	0,99	0,99 – 1,00
Desempleado	4.003	0,1	254	6,3	1,10	0,96 – 1,25
Desconocido	16.728	0,3	1.222	7,3	1,01	0,95 – 1,08
Área de Residencia						
Urbana^1^	5.833.681	89,2	338.817	5,8	1,00	-
Rural	703.710	10,8	35.942	5,1	0,87	0,86 – 0,88
Año de nacimiento					1,02	1,02 – 1,02

Fuente: Elaboración propia. OR= *Odds ratio.* IC95%= Intervalo de confianza del 95%. ^1^Valor de referencia.

## DISCUSIÓN

En Chile, los nacimientos prematuros han aumentado durante 1992 y 2018, presentando un promedio de porcentaje de cambio anual de 1,2. Sin embargo, la tendencia no ha sido constante durante el periodo, con una disminución en la primera mitad de la década de 1990, para aumentar progresivamente hasta la actualidad. A pesar de que existen importantes diferencias entre este aumento y la tendencia, se trata de un proceso similar a lo observado a nivel mundial, considerando el total de los partos prematuros^13^. Para un periodo similar, Australia mostró valores concordantes a los de este estudio, con un aumento de partos prematuros del 5,1% al 7,1%, asociando este fenómeno a nacimientos prematuros iatrogénicos, los que representaron el 80% de este aumento^15^. 

En cuanto a los resultados específicos por grupo de edad, este estudio muestra que, las mujeres mayores de 34 años presentan una probabilidad de parto prematuro más alta respecto a las mujeres más jóvenes. El comportamiento de la tendencia es similar en los tres grupos etarios, presentando dos puntos de cambio durante el periodo de estudio. Las mujeres menores de 35 años presentaron durante el primer periodo (1992-1994) una disminución estadísticamente significativa en la tasa de parto prematuro, mientras que las mujeres mayores de 34 años presentaron una tendencia más constante. Posterior a este periodo la tendencia ha aumentado significativamente en todos los grupos de edad con una magnitud similar. Estos resultados son consistentes con estudios previos en Chile^16^, en los que se evaluó la tendencia de parto prematuro entre los años 1991 a 2008, identificando que el riesgo de parto prematuro es mayor en los grupos de madres menores de 18 y mayores de 38 años. Asimismo, estudios en otras poblaciones muestran que la edad materna mayor a 35 años se asocia de forma independiente con resultados adversos en el embarazo, entre ellos el parto prematuro^17^. 

Aunque muchos factores sociodemográficos, nutricionales, biológicos y ambientales pueden aumentar el riesgo de parto prematuro espontáneo, la causa no se comprende por completo^18^. Respecto a los factores asociados, este estudio muestra que la chance de presentar un parto prematuro es mayor en aquellas mujeres de más de 35 años y que aumenta gradualmente mientras disminuye el nivel de estudios alcanzado. Un gradiente socioeconómico en el riesgo de parto prematuro se encuentra documentado en la evidencia, incluso en países con acceso universal a la atención prenatal^19^. El estudio de Knudsen *et al*. muestra un análisis de interacción aditiva entre la edad y las combinaciones de educación y condiciones de salud mental, señalando una interacción aditiva negativa con la edad ≤ 23 años y una interacción aditiva positiva con la edad ≥ 31 años. Esto indica que, con el aumento de la edad, el impacto de la educación y las condiciones de salud mental, tanto por separado como en combinación, son más importantes para el riesgo de parto prematuro. Además, concluyen que, para reducir la desigualdad en el parto prematuro, es esencial una atención centrada en las mujeres con mayor edad combinada con niveles educativos más bajos y condiciones de salud mental^19^.

Por otra parte, los resultados de este estudio para cada grupo de edad materna, en función del nivel educacional, mostraron que tanto las tasas de parto prematuro como el promedio del porcentaje del cambio anual son mayores en mujeres con educación básica de 35 años y más. Esto es consistente con lo señalado en la literatura: las madres que tienen un menor nivel de educación presentan un riesgo y tasas más elevadas de parto prematuro en comparación con aquellas que tienen mayor educación^20^^,^^21^.

El rol que juega la edad materna en el riesgo de un parto prematuro no está del todo claro. La literatura señala que el aumento de partos prematuros iatrogénicos se debe a complicaciones durante el embarazo tales como diabetes gestacional, hipertensión y restricción del crecimiento intrauterino. A su vez, dichas patologías son más frecuentes en mujeres de edad materna avanzada^22^. También se reporta que la incidencia de hipertensión durante el embarazo ha disminuido; sin embargo, entre aquellas diagnosticadas, el parto prematuro ha aumentado. Este es un aspecto relevante del problema, sobre todo si se considera que a nivel mundial ha habido una tendencia a postergar la maternidad por temas relativos a las trayectorias de las mujeres en la sociedad actual como el mercado laboral y académico^23^. 

Desafortunadamente, este estudio no brinda una explicación clara del aumento sostenido de estos nacimientos durante casi dos décadas en Chile y del rol que juega la edad materna. Esta limitación se debe principalmente a la falta de datos disponibles como aquellos relacionados con la atención obstétrica, antecedentes mórbidos, estilo de vida y contexto laboral de la gestante. Por otra parte, la fortaleza del presente estudio es haber obtenido los resultados sobre la base de la totalidad de los registros de nacimientos en Chile ocurridos en casi tres décadas y en el aporte de información.

Respecto de la calidad de los registros de los nacidos vivos en Chile cabe destacar que, para el año 1992, la oportunidad se estimaba cercana al 95%, aumentando a 99,8% en 2018. Esto se traduce en mejoras en la puntualidad de la inscripción de los nacimientos, y también en su completitud, dado que una mayor proporción de los nacimientos ocurridos cada año están siendo inscritos oportunamente^24^. Por otra parte, el alcance de la cobertura geográfica de los registros de nacimientos en Chile corresponde al total nacional, presentando una buena completitud de este registro, estimándose una omisión menor al 3% para el periodo en estudio^25^. 

Las implicancias del parto prematuro en términos de mortalidad infantil, de indicadores de calidad de atención materna y neonatal, así como de los costos asociados continúan siendo un problema de salud pública y hacen necesaria una mejor comprensión del problema^26^. Para ello es necesario mejorar la calidad y el volumen de los datos, incluida la estandarización de las definiciones, la medición y la notificación^5^. Se debe continuar con los esfuerzos para el logro de una de las metas propuestas del Objetivo de Desarrollo Sostenible 3, en cuanto a la reducción de las muertes evitables de recién nacidos y de niños menores de 5 años. Para ello es clave brindar asesoramiento clínico, otorgar vigilancia prenatal y realizar intervenciones de salud oportunas, sobre todo en madres cuyo riesgo de nacimiento prematuro es mayor. Además, es imprescindible desarrollar investigaciones que profundicen en los cambios epidemiológicos, transformaciones económicas y sociales que ha experimentado Chile durante las últimas décadas.
