# Supplementary material for: Tendencia temporal y factores asociados al parto prematuro en Chile, 1992-2018
Source: Salud Colect. 2023 Feb 1;19:e4203. [Article in Spanish] doi: 10.18294/sc.2023.4203 (PMC11930328; doi:10.18294/sc.2023.4203)
Supplement: Translation

# Translation: Temporal trends and factors associated with preterm birth in Chile, 1992-2018

**Authors:** Carol Toro-Huerta, Carolina Vidal, Luis Araya-Castillo

**Affiliations:** 1 Master in Public Policy. Instituto de Salud Pública, Universidad Andrés Bello. Santiago, Chile. carol.toro@unab.cl; 2 Master in Public Policy. Instituto de Salud Pública, Universidad Andrés Bello. Santiago, Chile. krolina.vidalg@gmail.com; 3 PhD in Mangement Sciences. Decano, Facultad de Ingeniería y Empresa, Universidad Católica Silva Henríquez, Santiago de Chile, Chile. larayac@ucsh.cl

**Keywords:** Premature Labor, Maternal Age, Maternal Health, Child Health, Chile.

## Abstract

An analytical study based on Chilean birth records obtained from the Department of Statistics and Health Information (DEIS) was conducted. This study aimed to evaluate temporal trends in preterm births by maternal age in Chile from 1990 to 2018. Results show that the preterm birth rate in 1992 was 5.0% and increased to 7.2% in 2018. The average annual percent change (AAPC) was 1.44. Age groups at the extremes (19 and under and 35 and over) presented the highest rates of preterm birth, both at the beginning and at the end of the study period. The latter group showed a smaller decrease at the beginning (1992 to 1995), with an annual percentage change (APC) of -3.00. The probability of preterm birth in both groups was higher compared to the 20-34 year old group. Although Chile boasts some of the best maternal and child health indicators in the region, repercussions associated with the current postponement of maternity - including preterm birth - must be monitored.

## INTRODUCTION

The World Health Organization (WHO) defines preterm birth as one that occurs before the 37^th^ week of pregnancy. Preterm birth continues to be a significant public health problem in many countries, as it has multiple consequences.^1^ On one hand, preterm newborns have a higher risk of morbidity and mortality during the neonatal period and long-term neurological and respiratory complications, as well as a higher risk of chronic diseases and psychiatric disorders in adulthood.^2^ On the other, mothers of preterm infants are more likely to develop depression and post-traumatic stress as compared to those who had a full-term delivery.^3^

The prevalence of preterm birth has increased in recent decades in most countries with reliable records.^4^ The global rate of preterm birth increased from 9.8% in 2000 to 10.6% in 2014, when there were an estimated 15 million preterm births. Regional variations were also observed, with rates of 13.4% and 8.7% for North Africa and Europe respectively.^5^

There are different causes of preterm birth, such as obstetric pathologies, infections, and maternal age of 35 and over.^6^ Evidence shows that advanced maternal age is associated with obstetric complications such as gestational diabetes, hypertension, and preeclampsia, as well as fetal complications such as intrauterine growth restriction and prematurity.^7^

In recent decades, there has been a tendency for women from high- and middle-income to increasingly postpone pregnancy. In the United States, the average age of first-time mothers rose from 24.2 in 2000 to 26.3 in 2014, while the number of first-time mothers aged 35 to 39 increased by 64% over the same period, and rose 230% among women aged 40 to 44.^8^

Chile has experienced changes in birth trends by age. A study by López in 2015 shows that the proportion of births among women aged 35 and over rose from 10.6% in 1991 to 16.6% in 2012. Additionally, the preterm birth rate increased from 4.7% to 6.4% in women aged 34 years and over during the same period, representing a 29% increase.^9^ Furthermore, López (2015) shows an odds ratio (OR) of 1.68 (95% CI 1.66 - 1.70) for preterm birth in mothers aged 35 or over, compared to 20- to 29-year-old mothers, adjusting for education, marital status, and parity.^9^

Reducing preterm birth rates is a global challenge to achieve Sustainable Development Goal 3, ensuring healthy lives and promoting well-being for all at all ages.^10^ Although Chile has good indicators of maternal and child health as a result of past policies, the slow development of preventive measures for preterm birth, the postponement of motherhood, and new technologies used in neonatal units must be taken into consideration to address this issue. Therefore, there is a need for an updated regional analysis regarding causes and at-risk groups in order to design strategies and policies.

With these considerations in mind, this study aimed to evaluate the temporal trends in preterm births by maternal age in Chile from 1990 to 2018. There are currently no up-to-date studies of trends in preterm births by maternal age that analyze long-term information.

## MATERIALS AND METHODS

An analytical study based on Chilean birth records obtained from the Department of Statistics and Health Information (DEIS) was conducted. Records contain information routinely extracted from birth certificates for all births in Chile during the period 1992-2018.^11^ The dataset includes sociodemographic and health characteristics of the child, parental demographics, and maternal factors associated with births.

Preterm births are defined by the WHO as those that occur before 37 weeks or 259 days of pregnancy from the first day of the last menstruation. It is subdivided into extremely preterm (less than 28 weeks), very preterm (28 to 32 weeks) and moderate or late preterm (32 to 37 weeks).^4^^,^^12^ Multiple births and stillbirths (less than 22 weeks gestational age and/or less than 500 grams) were excluded.

Annual rates of preterm birth in single births were calculated along with maternal age-specific rates according to three categories: 19 and under, 20 to 34, and 35 and over. To identify moments when significant changes in the trend occurred from 1992-2018 (as well as changes observed over the period), a *joinpoint* regression model was constructed (segmented Poisson regression). This model identifies the moments at which statistically significant changes in the trend occur, and estimates trends observed over the period based on average annual percent change (AAPC), a way of characterizing trends in rates over time. Based on the number of cases included in the analysis, a model was fitted with a maximum of 5 *joinpoints*. The program chooses the lowest number of *joinpoints* so that there is no statistically significant improvement if an additional *joinpoint* is added. The program starts with the minimum number of *joinpoints* (0), gradually adding new joinpoints by means of permutation tests until the final model is selected.^13^

Regarding mothers’ age groups, the trend was evaluated according to educational level: basic or elementary school, middle or high school, and higher education. For each group, AAPC was estimated using the *joinpoint* program, which represents a summary measure of the trend over the study period.

To assess the relationship between maternal age and preterm birth, a logistic regression model was used and odds ratios (OR) were calculated along with their 95% confidence intervals (95% CI). Adjustment variables included the sex of the newborn, birth year (to assess the trend), as well as the mother’s educational level, occupation, and area of residence.

This study used publicly available vital statistics records obtained from the Chilean Ministry of Health, which remained confidential and anonymous in compliance with Article 29 of Law 17,374.^14^

## RESULTS

Out of a total of 6,679,532 births registered from 1992 to 2018, 141,917 were excluded, leaving 6,537,615 single births to be included in the analysis. Of those excluded, 3250 correspond to deliveries of <500 g or <22 weeks, 124,290 were multiple deliveries, and 14,377 had missing data.

Figure 1 shows changes in the trend of preterm births over the study period. Results show that the preterm birth rate in 1992 was 5.0%, increasing to 7.2% in 2018; AAPC was 1.44 (95% CI [0.87, 2.01]). Three changes (*joinpoint*) in the trend were observed. In the first segment (1992-1994), a decrease in the trend was observed with an APC of -6.50 (95% CI [-11.33, -1.40]). In the second segment (1994-2006), an increase was observed with an APC of 2.86 (95% CI [2.49, 3.24]). Two other increases followed: between 2006 and 2010, with an APC of 0.18 (95% CI [-2.29, 2.72]), and between 2010 and 2018, with an APC of 2.02 (95% CI [1.48, 2.57]). All periods presented statistically significant changes, except for 2006-2010.

**Figure 1:** Time trend and points of change in the crude rate of preterm births, Chile 1992-2018.

Source: Authors’ own source. APC= Annual Percentage Changes. *Indicates that the Annual Percentage Change (APC) is significantly different from zero at the alpha = 0.05 level. Final Selected Model: 3 Joinpoints.

Figure 2 and Table 1 show changes in preterm birth trends by maternal age. For the 19 and under group, two *joinpoints* were observed. The first period (1992-1994) showed a decrease in preterm birth, with an APC of -7.50 (95% CI [-14.36, -0.09]); the second (1994-2005) and third period (2005-2018) showed statistically significant increases, with an APC of 2.09 (95% CI [1.47, 2.71]) and 1.06 (95% CI [0.61, 1.50]) respectively.

**Figure 2:** Temporal trend and points of change in the crude rate of preterm births by maternal age. Chile, 1992-2018.

Source: Own elaboration. APC = Annual Percent Change. *Indicates that the Annual Percent Change (APC) is significantly different from zero at the alpha = 0.05 level.

**Table 1:** Description of the number of joinpoints, annual percent change, and 95% confidence interval by maternal age. Chile, 1992-2018.

Age	Joinpoint	Lower endpoin	Upper endpoint	APC	95% CI
<19 years-old	2	1992	1994	-7.50*	-14.36; -0.09
		1994	2005	2.09*	1.47; 2.71
		2005	2018	1.06*	0.61; 1.50
20-34 years-old	2	1992	1994	-7.02*	-12.23; -1.43
		1994	2004	2.94*	2.37; 3.52
		2004	2018	1.68*	1.41; 1.94
> = 35 years-old	2	1992	1995	-3.00	-7.44; 1.66
		1995	2004	2.33*	1.36; 3.30
		2004	2018	1.19*	0.84; 1.54

Source: Own elaboration. APC = Annual Percent Change. *Significantly different from zero at the alpha = 0.05 level.

In the 20- to 34-year-old group, two *joinpoints* were observed in periods and magnitudes similar to those described in the previous age group, with an APC of -7.02 and two increases in the following periods: APC of 2.94 between 1994 and 2004, and 1.68 between 2004 and 2018.

Finally, in the 35 and over group, two *joinpoints* were also observed. The first period from 1992 to 1995 showed a decrease, although less significant than the one observed in the same period for the previous age groups, with an APC of -3.00. This was followed by two increases: 1995 to 2004 (APC of 2.33) and 2004 to 2018 (APC of 1.19).

The analysis incorporating average preterm birth APC between 1992 and 2018 taking into account the mother’s educational level showed an increase in average APC at each educational level for all age groups, and it was slightly higher at the basic educational level. According to these results, women aged 35 or over had the highest rates. In addition, the trend within this group was slightly higher in women with the lowest educational level, with an average APC of 2.03 (95% CI [1.73 - 2.33① (Table 2).

**Table 2:** Average Annual Percent Change (AAPC) for preterm birth, crude rate and 95% confidence interval by age and educational level of the mother. Chile, 1992-2018.

Variable	Crude rate		AAPC	95% CI
	1990	2018		
<19 years-old				
Basic or Elementary School	6.09	8.18	1.31	0.39 - 2.24
Middle or High School	5.60	7.59	0.89	0.18 - 1.60
Higher education	5.61	6.29	0.84	0.14 - 1.54
20-34 years-old				
Basic or Elementary School	4.62	7.57	2.07	1.49 - 2.65
Middle or High School	4.57	6.81	1.61	0.99 - 2.23
Higher education	4.32	6.42	2.00	1.62 - 2.30
> = 35 years-old				
Basic or Elementary School	6.70	9.88	2.03	1.73 - 2.33
Middle or High School	7.03	9.43	1.56	1.35 - 1.77
Higher education	6.40	8.37	1.24	1.00 - 1.49

Source: Own elaboration. AAPC = Average Annual Percent Change. 95% CI = 95% confidence interval.

The logistic regression model (Table 3) shows that the probability of preterm birth in women aged 35 years and over is 1.44 (95% CI [1.43; 1.45]) compared to the 20- to 34-year-old group, adjusted for the sex of the newborn, year of birth, mother’s educational level, occupation, and area of residence. Similarly, this probability was higher in adolescent mothers (<= 19 years-old).

**Table 3:** Odds ratios and 95% CI of the probability of preterm birth, by sociodemographic variables. Chile, 1992-2018.

Variables	Distribution of the sample		Preterm birth > = 22 weeks and < 37 weeks			
	n	%	n	%	OR	95% CI
Maternal age						
19 and under	940,485	14.4	57,808	6.1	1.19	1.18 – 1.20
20 to 34^1^	4,598,130	70.3	241,365	5.2	1.00	-
35 and over	998,215	15.3	75,554	7.6	1.44	1.43 – 1.45
Sex of the newborn						
Male	3,342,773	51.1	207,505	6.2	1.20	1.19 – 1.20
Female^1^	3,194,737	48.9	167,213	5.2	1.00	-
Educational level						
Higher Education^1^	1,498,242	22.9	89,228	6.0	1.00	-
Middle or High School	3,714,865	56.8	209.952	5.7	1.01	1.00 – 1.02
Basic or Elementary School	1,299,694	19.9	72,937	5.6	1.07	1.05 – 1.08
None	17,465	0.3	1,218	7.0	1.39	1.31 – 1.47
Occupation						
Inactive^1^	4,405,885	67.4	247,841	5.6	1.00	-
Active	2,110,988	32.3	125,466	5.9	0.99	0.99 – 1.00
Unemployed	4,003	0.1	254	6.3	1.10	0.96 – 1.25
Unknown	16,728	0.3	1,222	7.3	1.01	0.95 – 1.08
Area of residence						
Urban^1^	5,833,681	89.2	338,817	5.8	1.00	-
Rural	703,710	10.8	35,942	5.1	0.87	0.86 – 0.88

Source: Own elaboration. OR = Odds ratio. 95% CI = 95% Confidence interval. ^1^Reference category.

## DISCUSSION

In Chile, preterm births increased between 1992 and 2018, presenting an average annual percentage change of 1.2. However, the trend was not constant during this period and showed a decrease in the first half of the 1990s, followed by a gradual increase up to the present date. Although there are significant differences between this increase and the general trend, the pattern is similar to what is observed for the total number of preterm births worldwide.^12^ Over a similar period, comparable values to those observed in this study were reported in Australia, with preterm births increasing from 5.1% to 7.1%. This phenomenon was associated with iatrogenic preterm births, which accounted for 80% of the increase.^15^

Regarding specific results by age group, this study shows that women over 34 have a higher probability of preterm birth compared to younger women. This trend was observable in all three age groups, presenting two points of change over the period under study. Women under 35 showed a statistically significant decrease in the rate of preterm birth during the first period (1992-1994), while women over 34 showed a more constant trend. Following this period, trends significantly increased in all age groups similarly. 

These results are consistent with previous studies in Chile,^16^ where trends in preterm births were evaluated between 1991 and 2008, and it was shown that the risk of preterm birth was higher in mothers under 18 and over 38. Similarly, studies in other populations show that maternal age over 35 is independently associated with adverse pregnancy outcomes, including preterm birth.^17^

Although many sociodemographic, nutritional, biological, and environmental factors can increase the risk of spontaneous preterm birth, its causes are not yet fully understood.^18^ Regarding associated factors, this study shows that the risk of preterm birth is greater in women over 35 and increases gradually as education level decreases. A socioeconomic gradient in risk of preterm birth has been evidenced even in countries with universal access to prenatal care.^19^ A study conducted by Knudsen et al. showed an additive interaction analysis between age and the combination of education and mental health conditions, indicating a negative additive interaction with ages 23 and under and a positive additive interaction with ages 31 and over. This suggests that as age increases, the risk of preterm birth becomes more impacted by education and mental health conditions, both independently and in combination. The authors conclude that in order to reduce inequality in preterm births, it is essential to focus care on older women with lower educational levels and mental health conditions.^19^

On the other hand, the results of this study for each maternal age group by educational level showed that both preterm delivery and average APC rates are higher in women with basic education aged 35 years and older. This is consistent with the literature, as evidence suggests that mothers with lower education levels have higher risk and higher rates of preterm birth compared to those with higher education.^20^^,^^21^

The role of maternal age in the risk of preterm birth is not entirely clear. The literature shows that an increase in iatrogenic preterm birth is attributable to complications during pregnancy such as gestational diabetes, hypertension, and intrauterine growth restriction, and that these pathologies are more frequent in women of advanced maternal age.^22^ The decrease in the prevalence of hypertension during pregnancy has also been reported, although preterm birth has increased among those women diagnosed. This is relevant especially considering that there has been a global tendency to postpone childbearing due to issues related to women’s careers in today’s society such as participation in the labor market and academia.^23^

Unfortunately, this study does not provide a clear explanation for the sustained increase for nearly twenty years in preterm births in Chile, nor for the role of maternal age. This limitation is mainly due to the lack of available data related to obstetric care, clinical history, lifestyle, and work environment of pregnant women. Nonetheless, a strength of this study is the fact that its results were based on information extracted from Chilean birth records spanning nearly three decades.

Regarding the quality of live birth registration in Chile, it is worth noting that it was estimated at about 95% in 1992, increasing to 99.8% in 2018. This translates into improvements in the timeliness and completeness of birth registration, in the sense that a greater proportion of births are being registered each year in a timely manner.^24^ Furthermore, birth registries in Chile are national in their geographic coverage and have good levels of completeness, with an estimated omission rate of less than 3% for the period under study.^25^

The implications of preterm birth in terms of infant mortality, maternal and neonatal care quality indicators, and associated costs are still a public health concern that requires a better understanding.^26^ To that end, we need to improve data quality and volume, including standardization of definitions, measurement, and reporting.^5^ Efforts to achieve Sustainable Development Goal 3 regarding the reduction of preventable deaths of newborns and children under five must be sustained. To do so, it is crucial to provide clinical advice, prenatal surveillance, and timely health interventions, especially in mothers with higher risk of preterm birth. Furthermore, it is essential to carry out research that delves into the epidemiological changes and economic and social transformations that Chile has undergone in recent decades.
